# Microscale direct measurement of localized photothermal heating in tissue-mimetic hydrogels

**DOI:** 10.1038/s41598-019-42999-w

**Published:** 2019-04-25

**Authors:** Benyamin Davaji, James E. Richie, Chung Hoon Lee

**Affiliations:** 1 Electrical and Computer Engineering Department, Cornell University, Ithaca, NY USA; 2 Electrical and Computer Engineering Department, Marquette University, Milwaukee, WI USA

**Keywords:** Nanoparticles, Electrical and electronic engineering

## Abstract

Photothermal hyperthermia is proven to be an effective diagnostic tool for cancer therapy. The efficacy of this method directly relies on understanding the localization of the photothermal effect in the targeted region. Realizing the safe and effective concentration of nano-particles and the irradiation intensity and time requires spatiotemporal temperature monitoring during and after laser irradiation. Due to uniformities of the nanoparticle distribution and the complexities of the microenvironment, a direct temperature measurement in micro-scale is crucial for achieving precise thermal dose control. In this study, a 50 nm thin film nickel resistive temperature sensor was fabricated on a 300 nm SiN membrane to directly measure the local temperature variations of a hydrogel-GNR mixture under laser exposure with 2 mK temperature resolution. The chip-scale approach developed here is an effective tool to investigate localization of photothermal heating for hyperthermia applications for *in-vitro* and *ex-vivo* models. Considering the connection between thermal properties, porosity and the matrix stiffness in hydrogels, we present our results using the interplay between matrix stiffness of the hydrogel and its thermal properties: the stiffer the hydrogel, the higher the thermal conductivity resulting in lower photothermal heating. We measured 8.1, 7.4, and 5.6 °C temperature changes (from the room temperature, 20 °C) in hydrogel models with stiffness levels corresponding to adipose (4 kPa), muscle (13 kPa) and osteoid (30 kPa) tissues respectively by exposing them to 2 W/cm^2^ laser (808 nm) intensity for 150 seconds.

## Introduction

Hyperthermia cancer therapy (or tumor treatment) is commonly defined as the heating of the targeted cancer tissue in order to damage and induce apoptosis of cancer cells, or make them more susceptible to chemotherapy or trigger the immune system by thermal modulation^1,2^. The heating process can cleave the weak non-covalent hydrogen bonds and hydrophobic interactions between the base pairs. It can also selectively damage deoxyribonucleic acid (DNA) in cancer cells^3^, while avoiding the damage to the healthy tissue. Recently, it was shown that the application of hyperthermia can be extended to the subcellular domain^4^.

Hyperthermia cancer therapy can be achieved using the photothermal effect of plasmonic nanoparticles (NPs). The photothermal effect can be explained as the absorption of light by metal nanoparticles causing heat generation followed by dissipation of heat to the surrounding environment^5^. The light absorption of NPs can be maximized using surface plasmon resonance (SPR)^6^. The photothermal effect in NPs has been widely studied for nano-medicine and cancer therapy applications^7–9^. Photothermal hyperthermia can be used for the local heating of living tissue to enhance the delivery of specific drug carriers, as well as to improve the efficacy of therapeutic agents^2,10^. Localized NPs in targeted tissue can be used to convert the radiation of an electromagnetic wave into heat due to their photothermal properties. The key advantage of plasmonic NPs is to enable the localized heating of cancer cells^11^. Localized heat generation renders the therapy method minimally invasive^12^.

Gold NPs are the most widely used plasmonic NPs for light-induced hyperthermia therapy (also known as plasmonic photothermal therapy). Among GNPs, gold nanorods (GNRs) offer several advantages, including functionalization versatility, size-tunable optical characteristics, real-time imaging and distinct biocompatibility^13,14^. GNRs make plasmonic photothermal therapy (PPTT) more promising than other optical species due to their bio-targeting abilities, synthetic tunability, and large absorption cross-sections. GNRs have been widely explored as noncytotoxic photothermal agents with optical features in the biological window (NIR-I 690–850 nm)^5,15^. The fine-tunable longitudinal plasmon band of GNRs in the near-infrared (NIR, 0.75–1.4 *μ*m) region makes them suitable for therapeutic applications, as infrared light can penetrate into body tissues in this region^16^. There are several reports on the death of cancer cells in the presence of GNRs after exposure to NIR light *in-vitro* and *in-vivo*^17–20^. The geometry of GNRs alters the photothermal heat conversion factor and it can be used to tune the photothermal effect^21^.

The photothermal effect in NPs can be studied in either a dispersed^22,23^ or dry state^24^. Realizing precise insight into the photothermal heating effect requires investigation of the effect in real tissue or tissue-mimetic micro environments. It has been shown that hydrogels can effectively mimic the extracellular matrix (ECM) for cells^25,26^. The mechanical and thermal properties of ECM-like 3-dimensional matrices can be designed to model a real cellular environment in the human body. Precisely designed tissue mimetic hydrogel models for hyperthermia investigations will provide a pathway to reduce the expense of animal work to regulate the thermal dose for PPTT.

Precise temperature measurement is a key parameter to determine the localization of the temperature rise and effectiveness of PPTT. Many efforts in the past have been used to measure temperature using IR thermometers^27–30^. Thermographic systems are noninvasive temperature mapping techniques^24,31^ that detect thermal radiation from the surface of the sample^32^ with extended resolution down to micro-scale. IR thermometers have been commonly used in both *in-vivo* and *in-vitro* investigations of the photothermal effect^33–35^. In IR thermometry, the accuracy of measurements is disturbed by the differing emissivities and reflections from surrounding surfaces^36^ and is typically limited to bulk-scale measurements.

We present an on-chip planar contact micro-thermometer to measure laser-induced heating in GNRs dispersed in tissue-mimetic hydrogels. We provide quantitative measurement data for the photothermal effect of GNRs in the dry state, dispersed in a Phosphate-Buffered Saline (PBS) solution, and embedded in tissue-mimetic hydrogels. Using our microsensor, we investigate the impact of microenvironment thermal properties on the photothermal heating of GNRs. The presented data provides direct evidence that the microstructures within the hydrogel matrices govern the hyperthermia efficiency for localized heating. The developed device is a portable substrate which is compatible with any microscopy system and offers 0.002 K resolution for a sample volume as low as 200 nL.

**Figure 1:**
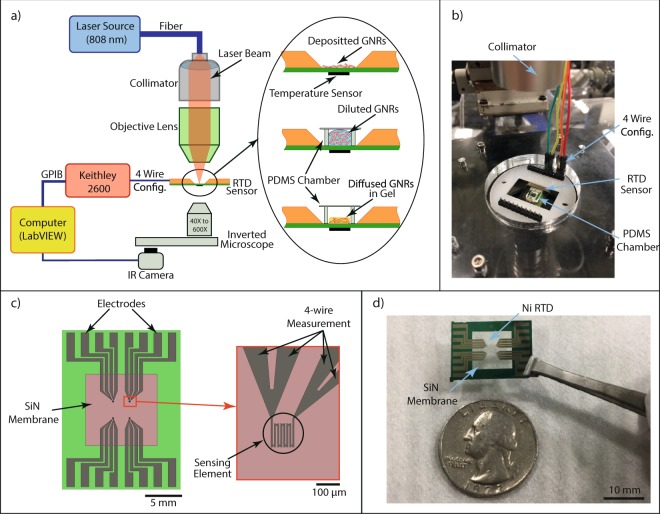
On-chip measurement setup for plasmonic photothermal heating at microscale. (**a**) Temperature measurement apparatus including: laser source, RTD temperature sensor, and inverted microscope. The resistance of the RTD sensor is measured by a 4-wire configuration using Keithley 2600 SMU controlled by a custom-made LabVIEW program. Inset shows three measurement configurations investigated in the results section: dried, liquid and gel. (**b**) A photograph of the measurement setup. (**c**) A schematic overview of our microsensor consisting of four nickel RTD temperature sensors on a SiN membrane, and a close-up view of devices shows the 100 × 100 *μ*m^2^ active sensing area of the RTD sensor. There are four sensing elements (RTD sensor) on each device. (**d**) A photograph of the on-chip RTD sensor developed in this work.

The on-chip measurement setup of GNR photothermal heating is shown in Fig. 1(a,b). The setup consists of three main parts: the continuous wave (CW) laser source and optical elements, a 4-wire resistance measurement microchip, and an inverted microscope. The CW laser source (808 nm) is coupled with an optical fiber (50, 100, and 400 *μ*m diameters, Thorlabs) to a collimator lens. The actual laser output power is measured and calibrated using a fiber-coupled reference power meter with 1 nW resolution (Thorlabs S142C). The collimated laser beam is focused with an objective lens on the region of interest to achieve desired spot sizes. A precision XYZ-stage manipulator is used to vary the focal point (z-axis) and location (xy-plane) of the beam. This setup allows the beam to be focused with a spot size as small as 50 *μ*m. The location of the laser spot to be varied within a 10 × 10 mm^2^ area on the device. A resistance temperature detector (RTD) sensor is used to directly measure the temperature of the sample. The RTD sensor was fabricated by thermal evaporation of a nickel film (~50 nm thick) onto a LPCVD deposited SiN membrane. The temperature sensor was configured to have high sensitivity with low thermal mass and high thermal isolation to the environment, see Fig. 1(c,d). The SiN membrane was fabricated by anisotropic wet chemical etch of silicon (30% w/w KOH solution at 60 °C) from the backside of the wafer. The nickel metal film was patterned in a serpentine shape to increase the sensing area and to set resistance at room temperature around 500 Ω, which showed an optimal sensitivity in this work. The fabrication process for the RTD sensor on SiN membrane is an standard micro-fabrication process^37,38^.

The experimental setup enables measurement of the photothermal heating on small samples (from 200 nL to 150 *μ*L). The achievable temperature resolution of our RTD is measured using a thermal bath setup to be 0.002 K^38,39^, which is higher compared to the thermal noise limit (Noise Equivalent Temperature) in RTD sensor (NET ~ 0.5 mK) and the resolution of our measurement system (SMU resolution ~ 0.4 mK). The resistance change of the sensor was measured in the 4-wire configuration using a Keithley 2600 source/measure unit (SMU)^40,41^. The measurements were performed at room temperature mainly to reduce the evaporation effect. Without any modification, the experiment can be performed at 37 °C for the biological samples. The resistance change is a function of temperature and can be expressed as, *R*_*T*_ = *R*_0_[1 + *α*(*T* − *T*_0_)], where *R*_*T*_ is the resistance at temperature *T*, *R*_0_ is the resistance at reference temperature *T*_0_, and *α* is the temperature coefficient of resistance (TCR). The TCR of the sensor is measured by a temperature-controlled calibration system between 10 to 50 °C. The measured TCR is 2.55 × 10^−3^ °C^−1^. The measured TCR of nickel film sensor is lower than TCR of a bulk nickel (6.8 × 10^−3^ °C^−1^). The lower TCR of deposited thin film is due to the grain size of the deposited film and the scattering due to film thickness^39^.

The sensor and sample were placed on an inverted microscope for visualization. The integrated IR camera to the inverted microscope (Fig. 1(a)) was used to locate the laser spot, align the beam with sample and sensor, and measure and control the laser spot size on the sample. Photothermal heating is characterized for GNRs in three states: dry, dispersed in PBS buffer, and in tissue-mimetic hydrogels. The heat generation in the dry state is a control measurement in comparison with the previously reported data^24^. GNRs dispersed in PBS are used to investigate the sample volume effect^42^. The results from the dry and dispersed in PBS states match the results of previously reported works and confirm the integrity of our measurement system.

For the first time, tissue-mimetic hydrogels have been used to imitate the human body tissue response to plasmonic photothermal heating using GNRs. In the dry state, a 50 *μ*L of the sample (dispersed GNRs in PBS) is dropped on the membrane and allowed to dry. Three different GNR densities (24, 56, and 280 ng/mm^2^) are used to measure the photothermal heating of GNRs in the dry state. In the dispersed state of GNRs in PBS, a Polydimethylsiloxane (PDMS) chamber was placed on the membrane to provide an isolated chamber for the liquid sample as shown in Fig. 1(a). PDMS is a silicon-based organic polymer, which is inert, non-toxic, and optically clear. Covering the chamber with a type #0 cover slip (~80 *μ*m thick) helps to prevent the evaporation of the media and decrease the heat dissipation to the environment. To investigate the sample volume (V) effect on photothermal heating, three different samples (20, 100, and 150 *μ*L) with a fixed concentration (N_GNR_ = 3.5 ng/*μ*L) were placed inside the PDMS chamber. For the gel state, a 6 × 6 mm^2^ of tissue-mimetic hydrogel containing different concentrations of GNRs was placed inside the PDMS chamber on the membrane. Detailed photothermal heating parameters are provided for three different hydrogels. Hydrogel stiffness values corresponding to adipose, muscle, and osteoid tissues are used. The effect of GNR concentration (7, 3.5, and 1.75 ng/*μ*L) on photothermal heating for each tissue-mimetic hydrogel is reported.

## Results

**Figure 2:**
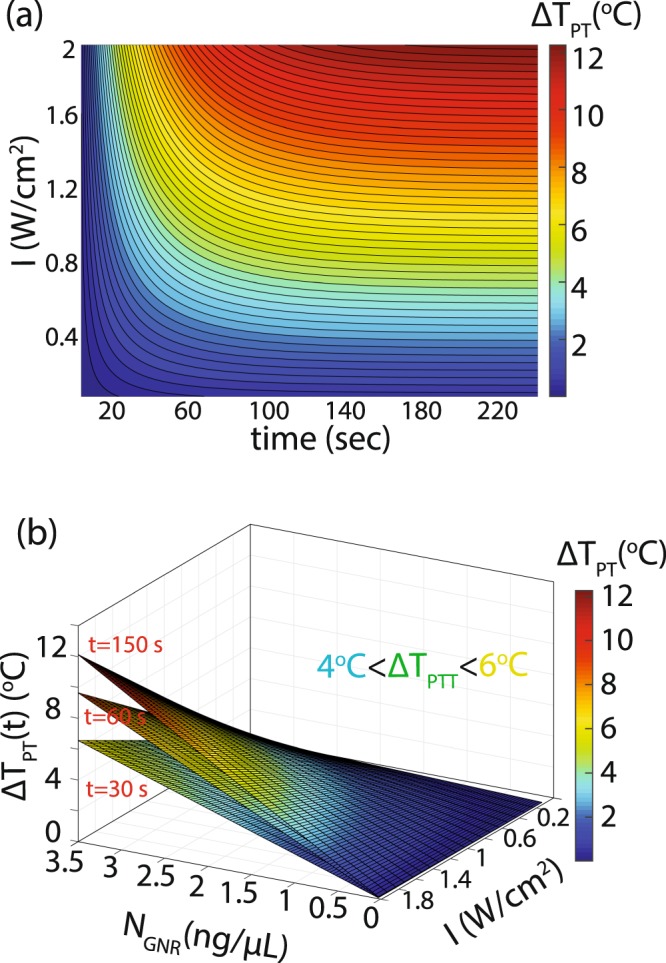
First-order estimation of the photothermal heating effect in a uniform distribution of GNRs. (**a**) For constant concentration of GNRs (3.5 ng/*μ*L), the green region of the surface plot shows the possible laser intensity and exposure time for PTT condition. (**b**) In real distributions the GNR concentration, laser intensity and exposure time will alter the photothermal effect (ΔT_PT_(x, y, z)) for each point in space.

Localized photothermal heating profiles are investigated for the three cases of solid powder (dried), dispersed in PBS solution (aqueous) and dispersed in Polyacrylamide (PAAm) hydrogels (gel). The dry state and aqueous state are control experiments and allow the interoperation of the measured profile in hydrogels accurately. The measurements in the dry state serve to confirm the functionality of our measurement by comparing the measured results with previously reported works^24^. The liquid state is a control measurement for calibrating the sample volume (V) effect. The GNR heating in a liquid state is a strong function of the sample volume^42^. We investigated the volume effect on photothermal heating by changing the volume of the dispersed GNRs in PBS from 20 *μ*L to 150 *μ*L. The thorough mixing of the GNRs with samples makes the uniform distribution an acceptable assumption in our model, however for real tissues the complex distribution of NPs should be considered. The photothermal heating (ΔT_PT_) can be estimated from the theoretical models^43,44^:

1 $${\rm{\Delta }}{T}_{PT}(t)={N}_{GNR}{c}_{abs}{I}_{laser}V{R}_{th}\mathrm{[1}-{e}^{t/{\tau }_{th}}]$$

where N_GNR_ is the concentration of GNRs, c_abs_ is the absorption cross section of the GNRs, I_laser_ is the intensity of the laser source and R_th_ is the thermal resistance of the system (microsensor material and geometry). The laser intensity and time can be controlled from outside to adjust the photothermal effect given uniform distribution of the GNRs in the substrate. The concentration of the GNRs also alters the photothermal effect and makes it complicated to calculate the heat distribution for the complex and an-isotropic substrates. Figure 2 shows a computed photothermal temperature change for a uniform distribution of GNRs. The trend estimated by these graphs provides the initial values for our experimental parameters.

PAAm hydrogels that mimic the extracellular matrix for cells were used to study the photothermal heating effect of GNRs in tissues. The effect of microstructure, porosity and stiffness of tissue-mimetic hydrogels on photothermal heating of GNRs was investigated to obtain insight regarding the accurate thermal dose for each one. Tuning the ratio of acrylamide monomer and bis-acrylamide cross-linker can change the porosity and elasticity of the PAAm hydrogel. In this investigation, three separate acrylamide/bis-acrylamide formulations were polymerized to yield hydrogels of 4, 13 and 30 kPa, which correspond to the stiffness of adipose tissue, muscle, and osteoid, respectively^45–47^.

### Photothermal response in dry state

**Figure 3:**
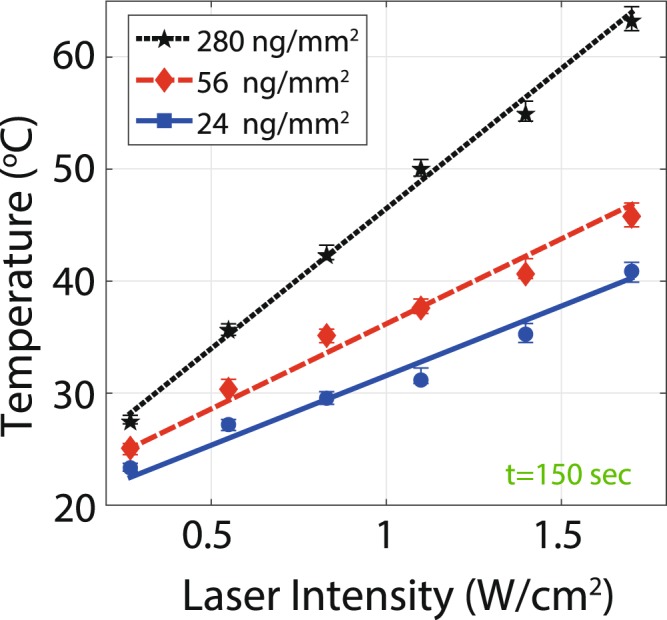
Photothermal temperature change of GNRs in the dry state after 150 sec exposure. The final photothermal temperature (T_PT_(t = 150 s)) is linearly proportional to the laser intensity.

Various amounts of GNRs are placed on the sensor (24, 56, and 280 ng/mm^2^) to investigate the effect of GNR density on heat generation. PBS with suspended GNRs were dropped by a metered pipette on the sensor area and allowed to dry. The temperature of the samples with respect to the laser intensity is shown in Fig. 3. The temperature increase (ΔT_PT_) is proportional to the surface density of GNRs. By increasing the surface density of GNRs from 24 to 280 ng/mm^2^, the final temperature at 1.7 W/cm^2^ laser intensity changes from 41.9 °C to 63.2 °C. We compare our results for GNRs in the dry state with previously reported work. In^24^, the photothermal heating of dried GNRs excited by a NIR laser is studied. They reported 35 °C temperature increase above ambient when the sample was exposed to 3 W/cm^2^ laser intensity for about 3 minutes. In our experiment, for the highest concentration, we measure about 43 °C above ambient when exposing the sample to 1.85 W/cm^2^ laser intensity for 150 s. This comparison confirms the functionality and reliability of our experimental setup. However, the difference in the results is due to the different GNR concentration, laser intensity, exposure time, and measurement method.

### Photothermal effect in liquid

We investigated the effect of volume on the photothermal heating by changing the volume of PBS containing GNRs from 20 *μ*L to 150 *μ*L (constant concentration). The dispersed GNRs in PBS (3.5 ng/*μ*L) are exposed to different intensities of the laser, in the range of 0.2–0.85 W/cm^2^ for 150 s. A differential measurement method was used to eliminate the temperature rise due to the laser absorption on the device membrane. The temperature change of the control sample (PBS only) was subtracted from the GNRs in PBS. The PBS is transparent to 808 nm light and has no direct effect on the photothermal temperature increase.

**Figure 4:**
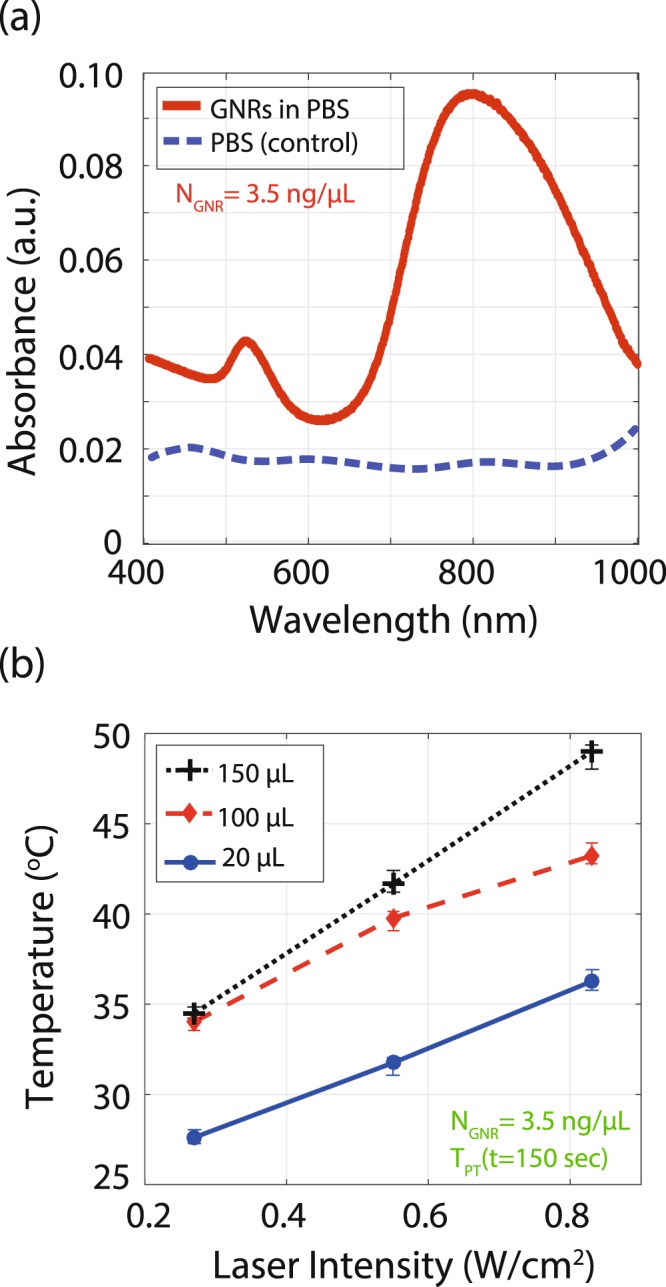
Photothermal effect of GNRs in PBS. (**a**) UV-Vis spectrum of GNRs in PBS. The dispersed GNRs in PBS have a maximum absorption at 808 nm wavelength. The PBS absorption spectrum shows a weak response without any resonance behaviour across the spectrum. (**b**) The steady state temperature (T_PT_(ss)) of the GNRs dispersed in PBS (volume: 20, 100, and 150 *μ*L with the same GNR concentration) for 150 s laser exposure.

The UV-visible spectrum of the dispersed GNRs in PBS has been measured as shown in Fig. 4(a). The results show that there is a strong absorption with GNRs in PBS and weak absorption without GNRs in PBS at 808 nm wavelength. The temperature (ambient is 20 °C) of the samples as a function of laser intensity are shown in Fig. 4(b). As shown in Fig. 4(b), the temperature of the sample increases proportionally as the laser intensity is increased and covers the photothermal therapy (PTT) window. The PTT window is defined as a 4 to 6 °C increase above the biological sample temperature (37 °C)^20,48,49^. Since we are using the synthetic substrate, the substrates are kept at room temperature to reduce evaporation. The 4 to 6 °C increase above room temperature is considered as an equivalent PTT window for our substrates. A larger sample volume (GNRs + PBS) results in a higher temperature increment. At a 0.85 W/cm^2^ laser intensity, samples of 20, 100, and 150 *μ*L each with the same concentration of GNRs show temperatures of 36.5, 43.2, and 48.9 °C, respectively. As the volume of the sample increases with constant GNR concentration and laser intensity, the amount of the heat generated increases due to additive temperature from the non-zero temperature tail of each NP. In other words, the heat profile from each GNR overlaps with the neighboring particle at microscale and the collective effect provides macroscopic heating and consequently a higher temperature change^42^.

### Photothermal heating of tissue-mimetic hydrogels

**Figure 5:**
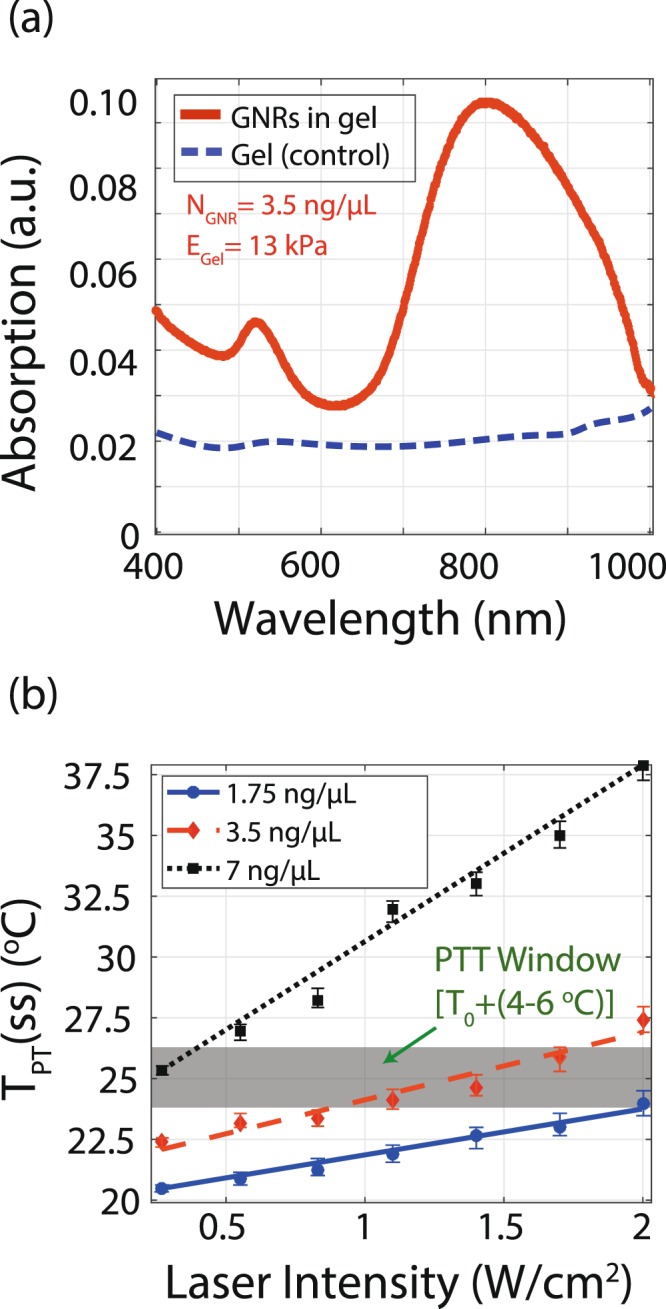
Photothermal heating of a medium (13 kPa) stiffness tissue-mimetic hydrogel with different GNR concentrations. (**a**) UV-Vis spectrum of GNRs dispersed in tissue-mimetic hydrogels. The hydrogel including GNRs shows a strong absorption at 808 nm wavelength. On the other hand, the hydrogel alone shows no absorption; (**b**) The steady state photothermal temperature (T_PT_(ss)) of GNRs dispersed in a tissue-mimetic hydrogel. The grey area indicates the photothermal therapy windows. Among the three concentrations of GNRs (1.75, 3.5, and 7 ng/*μ*L), the 3.5 ng/*μ*L concentration is in the range of the photothermal therapy window. The 3.5 ng/*μ*L GNR concentration is chosen for other hydrogel photothermal heating experiments.

In this section, the results of GNR photothermal heating in tissue-mimetic hydrogel models are presented. We studied different parameters on photothermal heating: concentration of GNR, laser intensity, exposure time, and hydrogel stiffness. The UV-Visible spectrophotometry of GNRs dispersed in hydrogel is shown in Fig. 5(a). As in PBS, the absorption behavior of GNRs is maintained after dispersion in the hydrogels. The hydrogel without GNRs shows a weak absorption at 808 nm wavelength.

#### The effect of concentration and laser intensity

The different concentrations (1.75, 3.5, and 7 ng/*μ*L) of GNRs dispersed in the synthesized hydrogel with medium stiffness (13 kPa) were exposed to the laser to find the best concentration fit for the PTT window (ΔT_PT_ = 4−6 °C). The temperature of each sample is shown in Fig. 5(b). From the measured data, the laser intensity as well as the GNR concentration are both directly proportional to the temperature increase of the tissue-mimetic hydrogels. At a 2 W/cm^2^ laser intensity, 1.75, 3.5, and 7 ng/*μ*L GNR concentration in the hydrogel (13 kPa, corresponds to stiffness muscle) shows 23.8, 27.5, and 37.7 °C temperature, respectively.

As shown in Fig. 5(b), the 3.5 ng/*μ*L GNR concentration in hydrogel is a good fit for the photothermal therapy window between 1 and 2 W/cm^2^ laser intensity. Therefore, we chose the GNR concentration of 3.5 ng/*μ*L for the remaining photothermal heating characterization.

#### The effect of thermal properties of hydrogel

**Figure 6:**
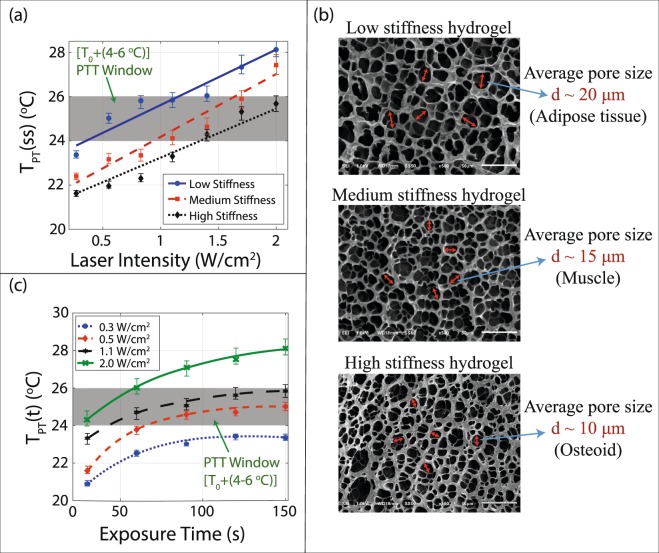
Photothermal heating of tissue-mimetic hydrogels with different stiffness. (**a**) Temperature increase (T_PT_(ss)) for three different stiffness hydrogels with 3.5 ng/*μ*L concentration of GNR as a function of laser intensity. The lowest hydrogel stiffness shows the highest temperature; (**b**) SEM micrograph of different stiffness hydrogels. The lower stiffness has the larger pore size between the gel structures. (**c**) Heating temperature (T_PT_(t), instantaneous temperature) increase of the lowest stiffness hydrogel as a function of the exposure time. The temperature tends to saturate after a certain exposure time.

Stiffness of hydrogel can be measured without going through the complex thermal analyses and yet can be used to represent the thermal properties of hydrogels (water content and the thermal conductivity of hydrogel). The photothermal heating results for tissue-mimetic hydrogels using a 3.5 ng/*μ*L GNR concentration are shown in Fig. 6(a). Changing the laser intensity (0.2–2 W/cm^2^) irradiated on the hydrogels (room temperature), caused the final temperature of the samples to increase from 21.7 to 28.1 °C with respect to the irradiation intensity. It is found that the hydrogels with lower stiffness (higher porosity) result in a higher temperature rise. As it is shown in Fig. 6(a), tuning of the laser intensity allows achieving the targeted temperature(ΔT_PT_) increase for each of hydrogels.

#### The effect of laser exposure time

The effect of exposure time at constant laser intensity on the hydrogels is investigated. As shown in Fig. 6(c), the temperature increases as a function of the exposure time. The raising temperature profile tends to saturate for each stiffness sample. It can be interpreted that temperature saturation is due to reaching a steady state condition where raising temperature profile due to the heat generation by photothermal effect and the heat dissipation to ambient settles to a final value. For the lowest stiffness hydrogel with a fixed laser intensity (1.1 W/cm^2^), the temperature of the sample varies from 23.4 to 25.9 °C in 150 s as shown in Fig. 6(c). For this hydrogel (4 kPa), the intensities of 0.5 to 1.1 W/cm^2^ are enough for achieving the temperature rise corresponding to the PTT window(4 °C < ΔT_PT_ < 6 °C).

## Discussion

The localized heating behavior based on plasmonic absorption in dispersed GNRs is a function of the concentration of GNRs in the media, the absorption cross section (c_abs_), and the laser intensity^50^. The absorption cross section has a direct effect on the heat generation and changes with the structural parameters of the GNRs. The absorption cross section of the GNRs for the three different states turns out to be constant in our experiments. For other photothermal heat generation dependencies, increasing the concentration of the GNRs and laser intensity are directly proportional to the heat generation and result in a higher temperature increase. We also investigated the effect of the sample volume on photothermal heating. Tuning the photothermal interaction volume allows us to simulate and study the photothermal heating effect for different volume sizes of targeted tissues and tumors. In our measurements we used the uniform distribution assumption for NPs in hydrogels, achieved by thorough mixing in our samples, however, to increase the accuracy of the model the effect of the local nonuniformities must be considered. The presented model can be adopted to predict the local photothermal heating for any realistic (nonuniform) distribution of NPs at steady state, while the transient behavior might need more careful considerations.

**Figure 7:**
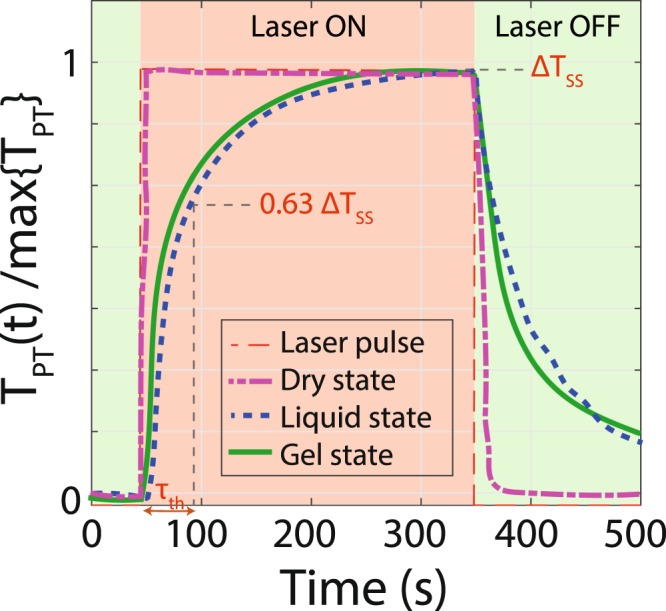
Temperature response time of different samples to laser pulse heating. The temperature of each sample is normalized to its maximum value for easy comparison. GNRs in the dry state have the fastest response. The gel state has a shorter time constant than that of liquid state. The difference in response times is due to the different thermal mass of the samples.

The temperature rise times (time constants) for powder (dried GNRs), in solution (GNRs in PBS) and dispersed in hydrogel (GNRs in gel) are compared in Fig. 7. GNRs in the dry state show a rapid increase in temperature and reach the steady state temperature during the laser exposure (dashed line). The quick rise of temperature (time constant < 1 ms) is due to fast (~ps) plasmonic heat generation^51^. The discrepancy between our measurement and the theoretical prediction of response time is due to the sampling rate of the measurement (~1 kHz) and finite thermal mass of the sensor. For dispersed GNRs in PBS, the time constant is about 40 s. The cooling profile follows Newtonian cooling when the laser is turned off (dotted line)^5,31^. The longer time constant compared with the dry sample is due to the higher thermal mass (C_th_) as a result of adding the PBS solution (C_th_ = C_GNR_ + C_PBS_ + C_Sensor_). For GNRs in hydrogel, the response is shorter than the response time of the liquid sample. The time constant for lowest stiffness hydrogel is about 30 s (solid line). The shorter time constant for gels compared to the liquid sample is due to the lower thermal resistance (R_th_) and higher thermal conductivity of the hydrogel^52^. Thermal time constant can be written as *τ* = C_th_ × R_th_, where C_th_ = C_GNR_ + C_Gel_ + C_Sensor_.

The thermal conductivity of the hydrogel can be modified by changing the porosity (cross-linking density) of the gel network while the hydrogel content stays almost unchanged^52–54^. Thus, in the case of unchanged content of the gel, the porosity is a key parameter that relates the stiffness to the thermal conductivity of hydrogels. The variations of the actual measured temperatures (error bars in Figs 5 and 6) in different samples directly corresponds to variations in substrate (deviation in gel thermal conductivity due to changes in porosity distribution and nonconformity of NPs distribution) and measurement conditions. Here, the specific heat of gels will be considered to stay almost unchanged. This assumption is acceptable for our first-order model; however, to increase the model accuracy the change in the specific heat of hydrogels should be considered. By controlling the porosity of hydrogel, thermal properties of various tissues can be mimicked and used in our temperature measurement platform to find an optimal condition for PPTT. The SEM micrographs in Fig. 6(b) show that higher stiffness hydrogels have smaller pore sizes (denoted as d in the figure).

The temperature profile modulated by the porosity of hydrogels is measured as shown in Fig. 6(a) and can be explained by the heat transfer through the hydrogel network. The larger hydrogel porosity (lower stiffness) results in a lower thermal conduction and heat generated at the surface of the GNRs in low stiffness hydrogel spreads out and dissipates to surrounding environment at a slower rate (easier to perform localized heating) compared to the stiffer hydrogels. The slower heat transfer is due to the lower thermal conductivity of the low stiffness hydrogel, and results in the higher local temperature increase. This observation suggests that the higher temperature change will be induced in tissues with lower stiffness at the constant laser intensity and the same GNR concentration level.

It is worth noting that for all of tested hydrogels, adjusting the laser intensity while staying in the reasonable range of laser power densities, allowed achieving the PTT window condition. We demonstrated the capability of our method and fabricated device to determine the laser intensity and exposure time required to achieve an efficient PPTT on various target tissue models. Additionally, our system is suitable for measuring the *ex-vivo* models, which can represent realistic concentrations and distribution of delivered nanoparticles to different volumes of tissues. The recently introduced graphene temperature sensors^55–57^ provide an untapped opportunity for moving toward applying this method to develop direct *in-vivo* measurements for localized photothermal effect and are yet to be investigated. In the future, we plan to map the temperature distribution across the sample by incorporating several sensors in an array fashion, integrated to a thin membrane. This microcalorimeter array will enable us to measure the photothermal effect and temperature gradient with high spatial resolution in the sample.

In this study, for the favorable penetration depth in tissue, we selected the photothermal agents (GNRs) in NIR-I biological window (lowest absorption coefficient for water and hemoglobin^58^). The presented technique is also applicable with other NPs operating in visible light^59^ or IR regions^60,61^ in spectrum. Finally, our developed system for localized photothermal investigations is compatible with other local hyperthermia techniques such as magnetic hyperthermia^62–64^, ultrasound hyperthermia^65,66^ and microwave hyperthermia^67,68^ with minor modifications. Also, this method can be modified to incorporate secondary effects like mechanical expansion and deformation because of photothermal heating by integrating a thin film stain sensor^69^.

We characterized the localized photothermal heating of GNRs dispersed in tissue-mimetic hydrogels, for the first time, using a direct temperature measurement. A microscale temperature sensor was developed to study the photothermal heating of the GNRs, directly and quantitatively in micro-scale volumes. The temperature profile of the tissue-mimetic hydrogels (4, 13, and 30 kPa stiffness which corresponds to the adipose tissue, muscle and osteoid, respectively) due to local photothermal heating was measured and characterized. The stiffness, as a practical measurable quantity for hydrogels, is used as an indirect indicator of thermal properties of hydrogels. The measured results show an inverse relationship between the stiffness of the hydrogels and the generated photothermal heat by laser excitation. The lower temperatures of photothermal therapy are therapeutically ineffective, and higher temperatures may cause adverse side effects. The developed platform and presented experimental procedure in this paper can be used to estimate the GNR concentration, laser intensity, and exposure time for effective and safe thermal doses for photothermal cancer therapy (inside the PTT window) in model hydrogels for mimicking different parts of the human body.

## Materials and Methods

### GNR plasmonic particles

We used 10 nm diameter GNRs (SPR 808 nm) from Nanopartz as the nano plasmonic agents for the localized heating. The sample was in a dispersed form in PBS and lower concentrations were made by diluting the original concentration with PBS.

### Tissue-mimetic Polyacrylamide hydrogels

The tissue-mimetic hydrogels were prepared as follows: Acrylamide, N, N methylene-bis-acrylamide, ammonium persulphate (APS) and N, N, N′, N′-tetramethyl -ethylenediamine (TEMED) were purchased from Sigma-Aldrich. A solution containing acrylamide monomers, crosslinker N, N methylene-bis-acrylamide, ammonium persulphate and N, N, N′, N′- tetramethylethylenediamine (TEMED) was prepared. Then various concentrations of GNRs dispersed in PBS were added to the prepared solution and mixed thoroughly. The prepared solution was poured inside a micromachined glass mold to make the desired thickness (300 *μ*m) of the Polyacrylamide (PAAm) hydrogels. Hydrogels with different stiffness were prepared to investigate the effect of the stiffness on the heat generation. The ratio of acrylamide%: bis-acrylamide% was varied (6:0.06, 10:0.1 and 10:0.3) in the process of gel making to adjust the hydrogel stiffness and porosity as reported elsewhere^70,71^.

### Stiffness of hydrogels

The hydrogels were made in high, medium, and low stiffness. To determine the stiffness of each hydrogel, the elastic modulus of the hydrogels were measured at 25 °C in compression mode using a universal testing machine (AGS-X series 100N, Shimadzu, Japan) at a speed of 1 mm/min. At least three samples of each hydrogel type were tested. The elastic moduli were derived from the initial linear region of the stress-strain curves.

### Hydrogel metrology

Micrographs of the freeze-dried hydrogels were taken using a scanning electron microscope (SEM) to analyze the microstructure and porosity of tissue-mimetic hydrogels. For the SEM imaging, the prepared hydrogels were gold sputtered using a GSL-1100X-SPC12 Compact Plasma Sputtering Coater instrument and imaged under SEM (JEOL JSM-6510LV). To determine the optical properties of GNRs dispersed in the hydrogels, all prepared hydrogels with and without GNRs were characterized using the spectrophotometry method (Thermo Scientific Evolution 220 UV-Visible Spectrophotometer) in the wavelength range from 400 to 1000 nm, with a resolution of 1 nm at room temperature.
